# MicroED in drug discovery

**DOI:** 10.1016/j.sbi.2023.102549

**Published:** 2023-02-21

**Authors:** Emma Danelius, Khushboo Patel, Brenda Gonzalez, Tamir Gonen

**Affiliations:** 1 Department of Biological Chemistry, University of California Los Angeles, 615 Charles E.Young Drive South, Los Angeles, CA 90095, USA; 2 Howard Hughes Medical Institute, University of California Los Angeles, Los Angeles, CA 90095, USA; 3 Department of Physiology, University of California Los Angeles, 615 Charles E. Young Drive South, Los Angeles, CA 90095, USA

**Keywords:** Cryo-EM, MicroED, Nanocrystals, Protein-ligand structures, Small molecule structures

## Abstract

The cryo-electron microscopy (cryo-EM) method microcrystal electron diffraction (MicroED) was initially described in 2013 and has recently gained attention as an emerging technique for research in drug discovery. As compared to other methods in structural biology, MicroED provides many advantages deriving from the use of nanocrystalline material for the investigations. Here, we review the recent advancements in the field of MicroED and show important examples of small molecule, peptide and protein structures that has contributed to the current development of this method as an important tool for drug discovery.

## Introduction

**Figure 1:**
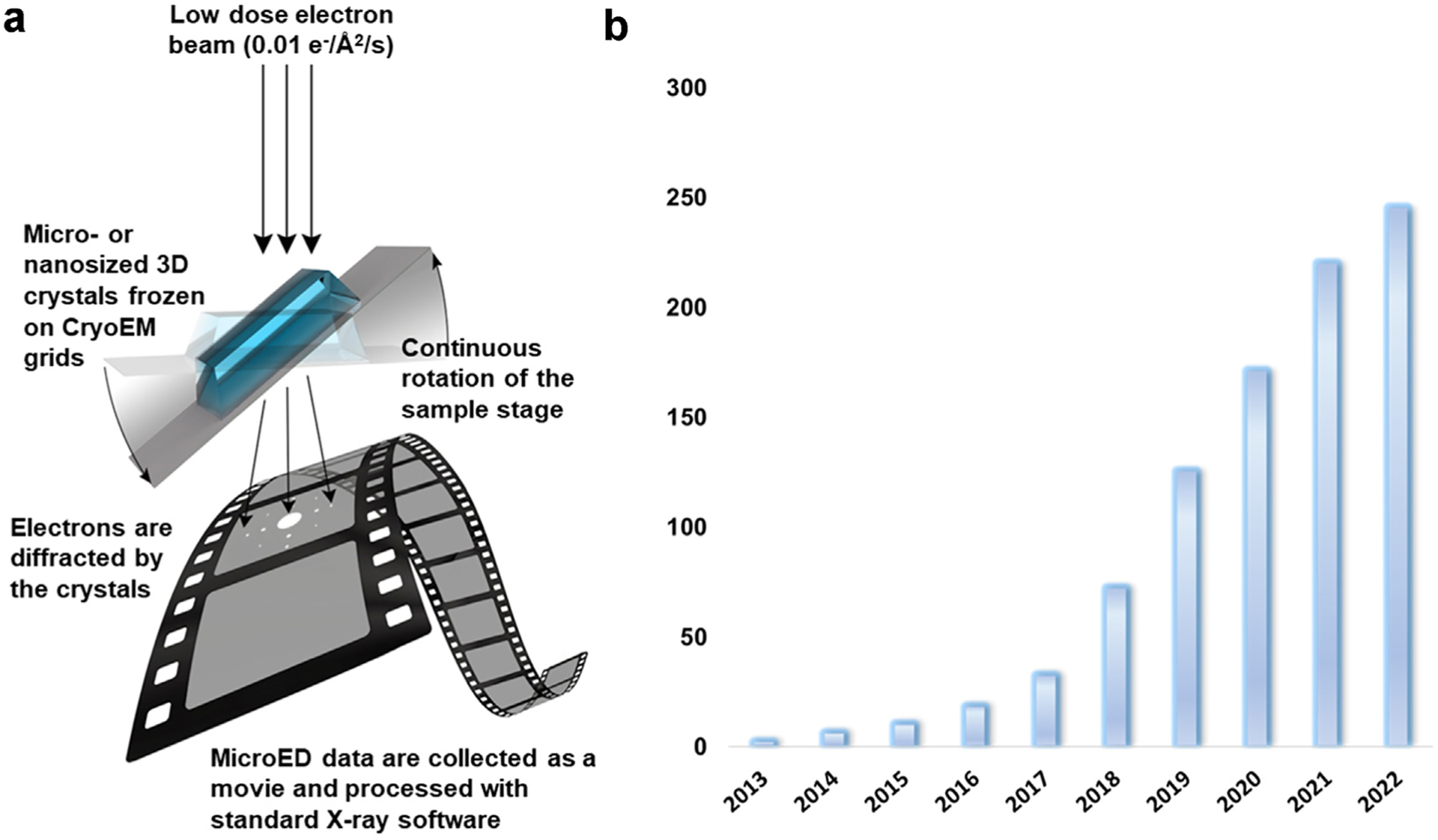
In MicroED, three-dimensional micro or nanosized crystals are continuously rotated in the electron beam in the TEM, and the data are collected on a high-speed camera as a movie. The data can be processed using the well-established X-ray diffraction programs. (B) Since its initial demonstration in 2013, the number of published MicroED structures have increased exponentially. The graph shows the total accumulated number of MicroED structures deposited in the pdb and CCDC databases as of the preparation of this figure.

**Figure 2:**
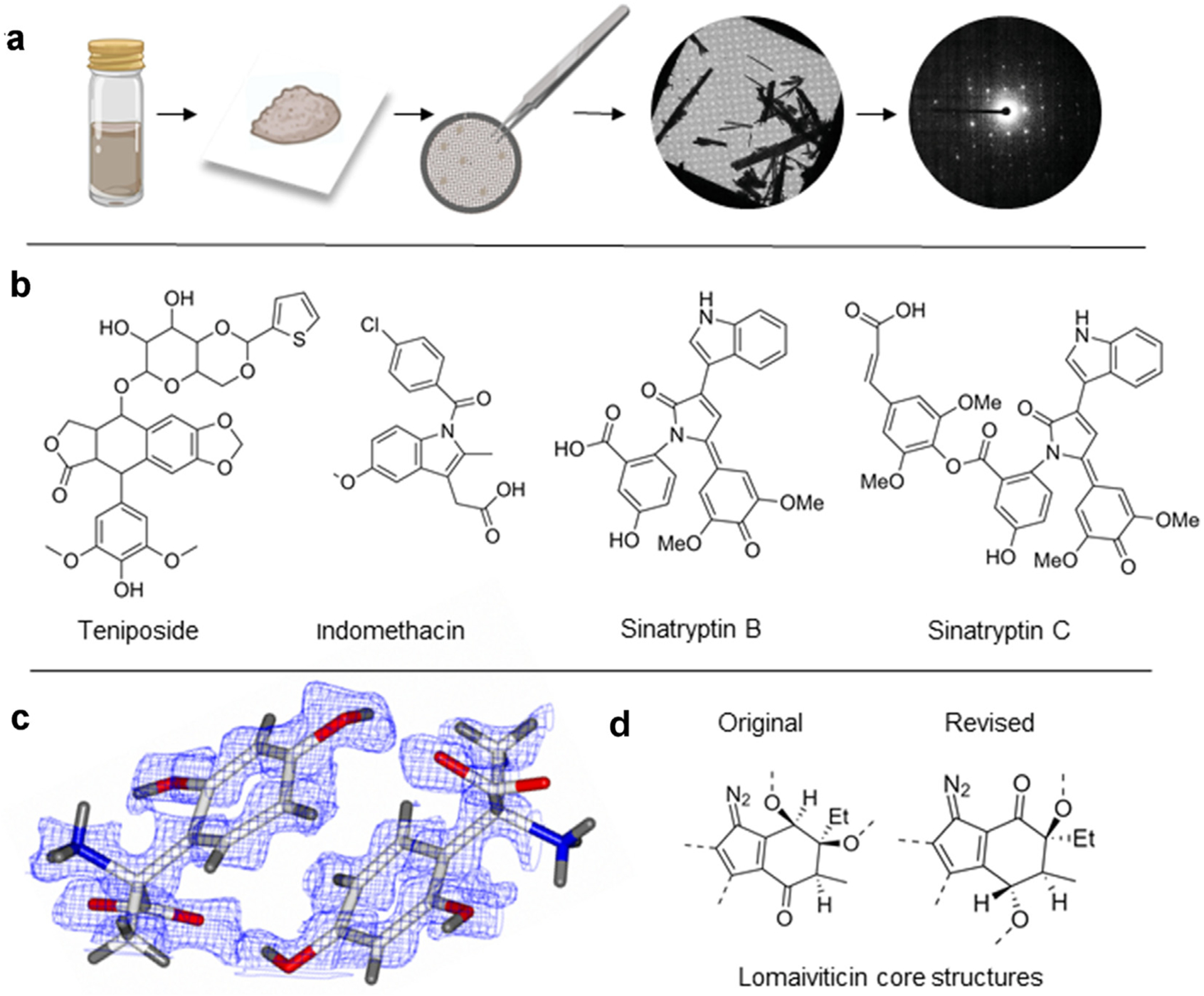
MicroED of small molecules: (a) MicroED workflow of small molecule structure determination directly from powder, without any prior crystallization step.(b) Recent pharmaceuticals and natural product structures determined by MicroED. (c) MicroED structure of 24DHPA with density map contoured at 1σ.(d) Structures of the original and revised assignment of the lomaiviticins core.

MicroED is an emerging technique in structural biology in which micro-or nanosized crystals are studied in the transmission electron microscope under cryogenic conditions (Figure 1a). MicroED has similarities to both cryo-EM and X-ray crystallography; the sample preparation and instrumentation resembles cryo-EM, whereas the data collection and processing is similar to X-ray crystallography [1]. Cryogenically freezing the sample is required to help reduce radiation damage [2]. After its initial demonstration on lysozyme in 2013 [3,4], and following a decade of technological and computational improvements leading to several published structures (Figure 2b), MicroED is now starting to find usefulness in the many areas of contemporary drug discovery. As compared to traditional structural methods, including X-ray crystallography and NMR, MicroED offers some important differances. For example, the crystals used in MicroED can be as small as a billionth the size of those used for X-ray diffraction and an atomic structure determined using just a femtogram amount of material. This has opened up the door for studying numerous important targets which were previously inaccessible due to crystallization difficulties. The use of nanocrystals in MicroED offers further advantages: ligand soaking procedures are expected to be more efficient and specialized *on-grid* soaking strategies are under development. Further, MicroED has shown great potential in advancing the field of membrane proteins, a class of proteins targeted by over 30% of the drugs on the global market, but only corresponding to about 2% of the unique structures in the PDB database. For small molecule drug candidates, MicroED can bypass the hurdles of any prior crystallization, and the structures are now commonly obtained directly from the powder material (Figure 2a). In addition, the powder used for the investigation does not have requirements on purity meaning mixtures can be studied and structure of different compounds determined from the same experiment, a unique feature of MicroED not possible by any other structural method. Here, we review the recent advancements in the field of MicroED and its relevance for drug discovery.

## Small molecule and natural product structures

The determination of structures of small molecules and natural products directly from powders by MicroED has been shown [5–11]. The powders were easily crushed between two glass cover slips, applied to carbon coated cryo-EM grids, and subjected to electron diffraction in TEM (Figure 2a). Following this initial proof of concept, mixtures of compounds were studied the same way, paving the way for new applications in natural product drug discovery and small molecule formulation. Interestingly, solid-state grinding of powder mixtures has been shown to provide “mechano-distinctive” co-crystals that are not accessible from solutions [12]. In the last couple of years, the number of small molecules and natural product structures determined using MicroED has increased exponentially. One of the major contributions was the development of a MicroED pipeline for determination of small molecule structures at industrial level by Bruhn et al. They were able to successfully determine the structure of the novel compound teniposide (Figure 2b), followed by more than 50 structures of small molecules for which producing single crystals for x-ray diffraction was nearly impossible [13]. Another important contribution was the structure of a third polymorph of the widely used non-steroidal anti-inflammatory drug indomethacin (Figure 2b) which was solved by MicroED [14,15]. In this case, producing large single crystals had been explored without success since its discovery in 1974.

The use of MicroED for determining configuration was initially demonstrated in 2019 [16], and further validated on active pharmaceutical ingredients (APIs) by comparing to previously described X-ray structures, where they were found to be exactly same [17]. The configuration of unnatural amino acid intermediates has also been examined, for example, the 0.62 Å structure of the enzymatic reaction product 2-amino-2-(2,4-dihydroxyphenyl)propanoic acid (24DHPA) was solved by MicroED in as minimal time as 1 h (Figure 2c) [18]. The obtained structure confirmed the presence of en-antiomers that were only speculated by NMR, MS and circular dichroism.

MicroED has also been useful in advancing the research in natural products and metabolite discovery. The structure of a *Caenorhabditis elegans N*^3^-(β-glucopyranosyl) uric acid metabolite was obtained using MicroED in combination with organic synthesis [19]. Further, the structures of eight novel algicides produced by marine bacterial symbiont *Phaeobacter inhibens*, including sina-tryptin B and C (Figure 2b), were determined from microcrystals grown on grid by slow evaporation, where three of the compounds were produced in quantities as low as 0.2e0.5 mg [20]. Kim et al. contributed the novel structure of lomaiviticins and thereby cleared the decade long confusion of placement of the bridging carbonecarbon bond and the orientation of the cyclohexane ring and secondary glycoside (Figure 2d) [21]. These genotoxic metabolites contain only 6 carbon atoms attached to protons in a monomeric aglycon unit which makes it challenging for structural determination using NMR. For the natural product fischerin, discovered over two decades ago, MicroED was useful in determining correct stereochemistry while NMR and X-ray crystallography were proven futile [22]. Additionally, during the data collection from fischerin microcrystals, the authors noticed another crystalline impurity that was undetected by solution state NMR. Their structural determination of byproduct austinol from the same experiment demonstrates the sensitivity of MicroED for quantities to as low as 3 ng, as well as the advantage of being able to use impure products or mixtures. Additionally, automation software, such as SerialEM [23–25], are under evaluation for MicroED and are expected to further advance this technique in the field of small molecule and natural product in drug discovery.

## Ligand-bound protein structures

**Figure 3:**
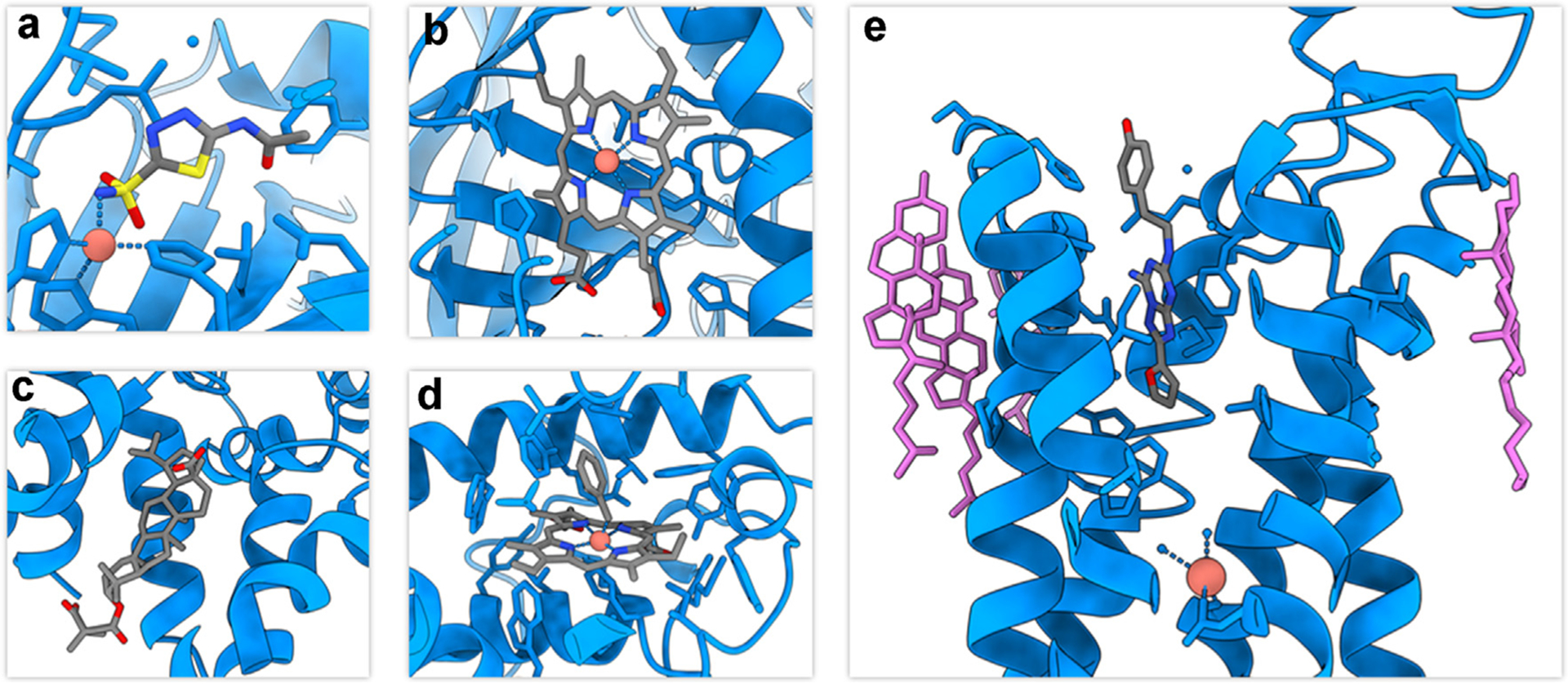
Examples of ligand-bound protein structures solved by MicroED: a. The HCA II–AZM binding site from a 2.5 Å resolution MicroED structure [29]. b. The3.2 Å structure of catalase solved by MicroED, showing the heme binding site [37]. c. The MicroED structure of the CTD-SP1 fragment of HIV-1 Gag with bound maturation inhibitor bevirimat [38]. d. The 2.5 Å MicroED structure of a protoglobin reactive carbene intermediate at the heme binding site [39]. e. MicroED structure of the human adenosine receptor at 2.8 Å, showing the binding site of the antagonist ZMA (grey) as well as several cholesterols binding to the membrane helices (pink) [35].

MicroED has recently gained momentum in the determination of novel structures as well as ligand-bound complexes, which are both important for structure-based drug design and fragment screening. Since atomic resolution data can be obtained from nanocrystals less than 300 nm in thickness [26], MicroED has potential to be the main approach for ligand soaking experiments. Diffusion of small molecules into tiny crystals is extremely efficient allowing for shorter soaking times as compared to the large crystals needed for X-ray diffraction, leading to less crystal damage and loss of high-resolution data. This was evaluated using the enzyme proteinase K and the I3C ligand [27]. Martynowycz et al. demonstrated the feasibility of on-grid soaking of protein and obtained a 1.78 Å structure of the protein-ligand complex. The occupancy of the ligands was improved as compared to the X-ray study [28], and an additional binding site was identified. In another ligand soaking study, MicroED was used to describe the 2.5 Å structure of human carbonic anhydrase isoform II (HCA II) bound to the sulfonamide inhibitor acetazolamide (AZM, Figure 3a) [29], to comparable resolution as the already described X-ray structure [30]. These initial reports suggested that ligand soaking in MicroED is a promising method for obtaining information for structural based drug design.

## Membrane proteins and FIB-milling

Membrane proteins are notoriously hard to crystallize, as illustrated by the very few examples derived by X-ray diffraction [31], and the majority of the drugs targeting this important class of proteins have been developed without any structural information. Moreover, most membrane proteins are too small for imaging by single particle cryo-EM so they remain in the domain of crystallography. Luckily, membrane proteins often form nanocrystals which might be useful for MicroED. Following the initial reports of the Calcium ATPase [32], NaK ion channel [33] and the voltage-dependent anion channel VDAC [34], MicroED has recently been successfully used to study membrane proteins in lipidic cubic phase (LCP), providing a closer to native environment for the investigations. The structure of the antagonist-bound human A2A adenosine receptor (Figure 3e) was determined from crystals prepared in the jelly-like LCP [35]. The crystals were milled to an appropriate thickness using a focus ion beam (FIB) and the final structure was obtained from a single milled-lamella only 200 nm thick. In addition to the receptor-bound ligand, the binding sites of four cholesterol molecules were identified (Figure 3e). Using MicroED, it was also possible to identify four disulfide bridges in the density map, which was not possible with X-ray diffraction due to radiation damage. The most recent improvements include fluorescently labeling the crystals to easily identify them in the LCP using correlative light and electron microscopy (CLEM), in this case further improving the resolution of the A2A receptor to 2Å [36].

## Phasing strategies and solving novel structures

**Figure 4:**
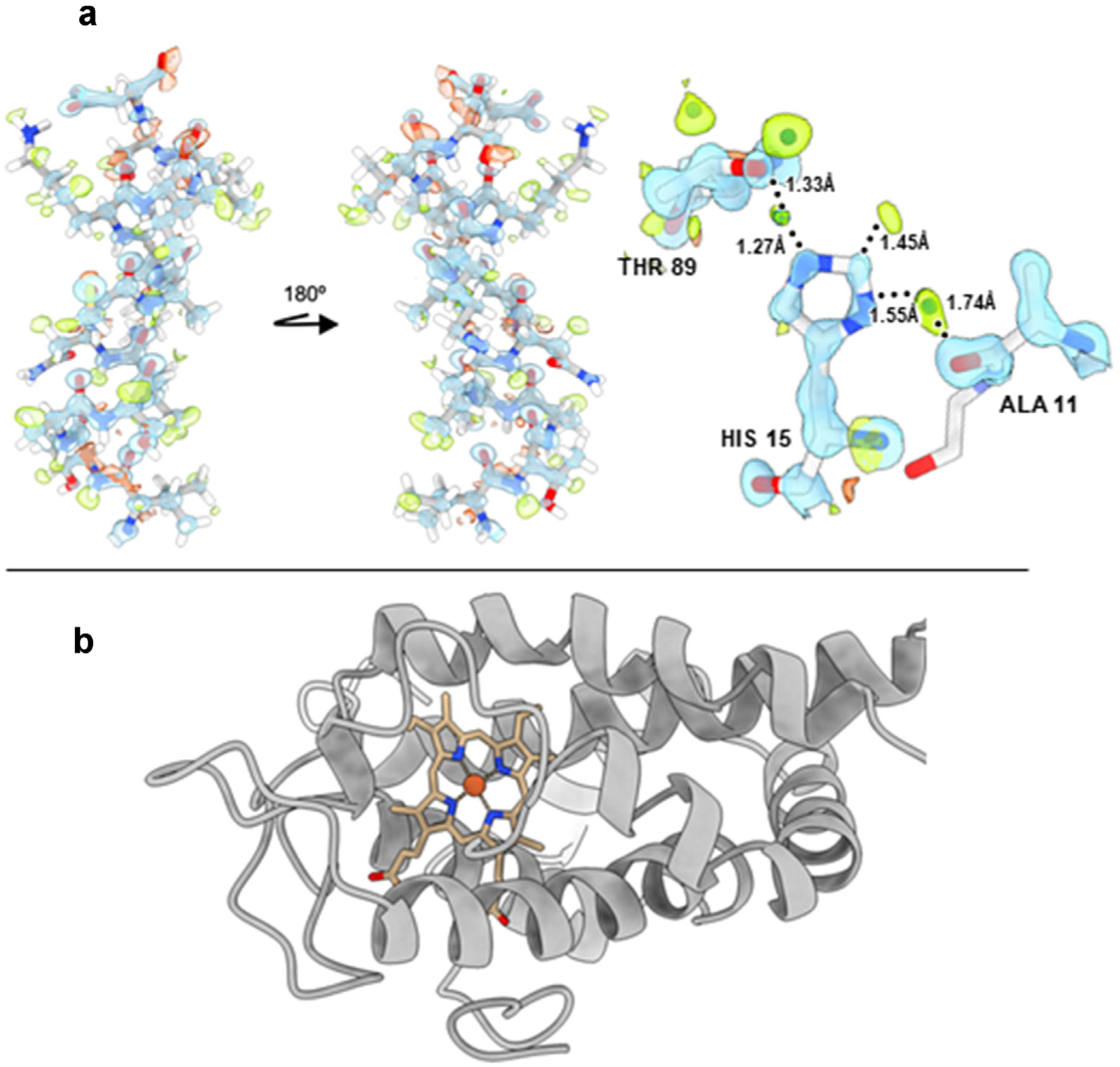
Phasing in MicroED: a. The 0.87 Å resolution structure of lysozyme was determined with ab *initio* phasing. This sub-Ångstrom structure showed density for hydrogens (displayed in green) as well revealed the histidine 15 to be charged, as shown here in the close-up of this residue with the corresponding hydrogen bonds to alanine 11 and threonine 89 [41]. b. The novel structure of a protoglobin *Aeropyrum pernix protoglobin* engineered for carbene transfer reactions in asymmetric synthesis. No wild-type homologue was available and the structure was determined using an AlphaFold2-predicted structure for phasing of the MicroED data [42].

Although there are now over 120 unique MicroED entries in the PDB (Figure 1), the contribution of novel structures has so far been scarce. Similar to X-ray diffraction, the phase information in MicroED is lost during data collection and has to be solved in order to determine the structures. For resolutions better than 1 Å the phases can be estimated directly from the intensities through *ab initio* methods, and this is the typical case for small molecule structural determination. One of the major breakthrough structure determination by MicroED was the recent report of a protein structure determined *ab initio*. Using FIB milling and electron counting with a direct electron detector for MicroED data collection, the sub-Ångstrom resolution structure of triclinic lysozyme was determined with *ab initio* phasing (Figure 4a) [40]. Structural information at this resolution allows for visualizing the hydrogen bods and the charged state of the residues (Figure 4) [41], both important concepts in structural based drug discovery. As with X-ray crystallography, molecular replacement is the most common method of solving the phase problem of protein samples. Here, the phase information is derived from a related homologous protein with known structure. Taking advantage of the advancements in structure prediction, a recent report shows that MicroED structures now can be solved even in the absence of structures of related homologues. The novel structure of *Aeropyrum pernix* protoglobin (*Ape*Pgb, Figure 4b) was determined using an *ab initio* predicted model from AlphaFold2 for phasing [42]. Moreover, the reactive intermediate was also captured by MicroED allowing a first look into a carbene-metal reactive intermediate [39]. This shows that previously unachievable structures of new and important drug targets can now be obtained from nanosized crystals in combination with structure prediction.

## Conclusion

MicroED opens up many opportunities to move forward difficult projects in drug discovery. Here we briefly described the advantages of using MicroED for studying small molecule drug candidates, natural products, ligandeprotein complexes and membrane proteins for drug discovery applications. With the recent technological advancements in the field leading to very high-resolution structures, novel protein-ligand complexes, and many new or revised small molecule structures, MicroED has shown that it can contribute key information and has the potential to be an important method for future drug discovery.

## Data Availability

No data was used for the research described in the article.
